# GABA Neuron Alterations, Cortical Circuit Dysfunction and Cognitive Deficits in Schizophrenia

**DOI:** 10.1155/2011/723184

**Published:** 2011-09-05

**Authors:** Guillermo Gonzalez-Burgos, Kenneth N. Fish, David A. Lewis

**Affiliations:** Translational Neuroscience Program, Department of Psychiatry, University of Pittsburgh School of Medicine, Pittsburgh, PA 15261, USA

## Abstract

Schizophrenia is a brain disorder associated with cognitive deficits that severely affect the patients' capacity for daily functioning. Whereas our understanding of its pathophysiology is limited, postmortem studies suggest that schizophrenia is associated with deficits of GABA-mediated synaptic transmission. A major role of GABA-mediated transmission may be producing synchronized network oscillations which are currently hypothesized to be essential for normal cognitive function. Therefore, cognitive deficits in schizophrenia may result from a GABA synapse dysfunction that disturbs neural synchrony. Here, we highlight recent studies further suggesting alterations of GABA transmission and network oscillations in schizophrenia. We also review current models for the mechanisms of GABA-mediated synchronization of neural activity, focusing on parvalbumin-positive GABA neurons, which are altered in schizophrenia and whose function has been strongly linked to the production of neural synchrony. Alterations of GABA signaling that impair gamma oscillations and, as a result, cognitive function suggest paths for novel therapeutic interventions.

## 1. Introduction

Schizophrenia is a severe brain disorder that afflicts 0.5–1% of the world's population and that is typically first diagnosed in late adolescence or early adulthood. The illness is manifest as disturbances in perception, attention, volition, inferential thinking, fluency and production of language, and the recognition and expression of emotion that lead to substantial impairments in social and occupational functioning. Many affected individuals suffer from comorbid depression, an increased risk of cardiovascular disease, and excessive nicotine, alcohol, and cannabis use. 

Three major domains of symptoms define schizophrenia. The first domain is positive or psychotic symptoms that include delusions, false beliefs firmly held in the face of contradictory evidence; perceptual disturbances and hallucinations, which may occur in any sensory modality but are most commonly auditory and experienced as hearing voices distinct from one's own thoughts; abnormalities in form of thought that are usually manifest as loose associations, over-inclusiveness, and/or neologisms; abnormal psychomotor activity that is usually manifest as grossly disorganized behavior, posturing, and/or catatonia. Negative symptoms include asociality (withdrawal or isolation from family and friends), avolition (impaired initiative, motivation, and decision-making), alogia (poverty in the amount or content of speech), and anhedonia (reduced capacity to experience pleasure). The third category of symptoms includes a number of cognitive abnormalities such as disturbances in selective attention, working memory, executive control, episodic memory, language comprehension, and social-emotional processing. 

Although positive symptoms are usually the presenting and most striking clinical feature of schizophrenia, disturbances in cognition appear to be the core features of the illness as they are present before the onset of psychosis and are the best predictor of long-term functional outcome for schizophrenia patients [1]. Therefore, functional recovery (e.g., recovery of the capacity to maintain employment) is largely dependent on improving cognitive deficits. 

Although schizophrenia was initially characterized over 100 years ago, we still have only a limited understanding of its pathophysiology. Moreover, we lack efficient tools for its treatment or prevention. For example, the multicenter, NIMH-funded Clinical Antipsychotic Trials in Intervention Effectiveness project recently found that newer atypical antipsychotics are not significantly more effective for treating psychosis than older typical antipsychotic medications and showed little benefit for improving cognitive symptoms [2, 3]. These findings highlight the need to develop novel therapeutic interventions for schizophrenia [4, 5]. 

If functional recovery of patients with schizophrenia depends on improving cognitive deficits, then understanding the neural basis of the normal cognitive operations that are impaired in schizophrenia is crucial to develop new therapies. Interestingly, a number of findings from postmortem brain studies suggest that schizophrenia is associated with deficits of GABA-mediated synaptic transmission [6]. Furthermore, current hypotheses from cellular and systems neurophysiology suggest that a major role of GABA-mediated transmission is to produce synchronized neural network oscillations [7, 8] which by facilitating the processing and flow of information within and between brain regions may be essential for normal cognitive function [9]. Here we review convergent findings from schizophrenia research, cellular neurophysiology, and cognitive neuroscience that favor the hypothesis that deficits of cognitive function in schizophrenia result from a dysfunction in GABA-mediated synaptic inhibition that disturbs oscillatory neural synchrony. 

This paper reviews recent evidence further indicating that in subjects with schizophrenia cognitive dysfunction is associated with alterations of oscillations in the gamma frequency band (30–80 Hz), which are normally induced during tasks that engage cognition [10]. In addition, it reviews the cellular and molecular machinery involved in GABA-mediated synaptic transmission and the mechanisms by which GABA-mediated inhibition may synchronize neural activity in cortical circuits, focusing on the role of parvalbumin- (PV-) positive GABA neurons, whose function has been increasingly linked to the production of synchronized gamma oscillations. Furthermore, data on the postnatal development of PV GABA neurons and their synaptic connections and the developmental trajectories of gene products involved in GABA-mediated synaptic inhibition is reviewed. Finally, recent studies further suggesting that schizophrenia is associated with alterations in GABA-mediated synaptic transmission, particularly, but not exclusively, from PV neurons are highlighted. Alterations of GABA signaling that impair gamma oscillations and therefore cognitive function in schizophrenia suggest potential paths for therapeutic interventions.

## 2. Altered Neural Synchrony and CognitiveFunction in Schizophrenia

Schizophrenia is associated with deficits in behavioral tasks that assess perceptual and cognitive processes [10, 11]. Many such tasks normally increase synchronized oscillatory activity as measured in the electroencephalogram (EEG), and such increase in synchrony is altered in subjects with schizophrenia [11, 12]. For example, gamma-band synchrony during tasks that require visual gestalt perception is attenuated in schizophrenia patients [13]. One of the core cognitive deficits in schizophrenia is a dysfunction of working memory, a system to keep information in mind and to manipulate it while performing complex tasks [14]. Gamma oscillatory activity (30–80 Hz) may play an important role in normal working memory, given that gamma band synchrony increases with increasing working memory load [15, 16]. In patients with schizophrenia, working memory deficits are accompanied by altered patterns of cortical oscillatory activity [11], since schizophrenia patients actually fail to enhance gamma activity with increasing working memory load [16] and show overall increased gamma band power during working memory [16, 17]. Subjects with schizophrenia also have decreased oscillations in various frequency bands during specific phases of the working memory process, including encoding, maintenance, and retrieval [18]. 

Cognitive function, including working memory, implicates an interconnected network of brain regions, many of which show structural and functional abnormalities in schizophrenia [19]. The prefrontal cortex (PFC), which is extensively interconnected with cortical and subcortical regions, is thought to exert top-down control of the flow of neural activity between brain regions to provide cognitive control, coordinating incoming sensory and motor information with representations of internal goals and rules to select a context-appropriate behavioral response [20]. Subjects with schizophrenia have significant deficits in cognitive control [10] and attenuated gamma oscillations in PFC during cognitive control tasks [21]. Cognitive control-related gamma activity, but not theta activity, is reduced in the frontal cortex of first-episode schizophrenia patients independent of medication status, suggesting a deficit related to the disease process as opposed to medication side effects or the consequences of being chronically ill [22]. Interestingly, some studies reported a positive correlation between gamma oscillations and hallucination symptoms score in schizophrenia [23, 24], indicating that the propensity for auditory hallucinations correlates with an increased tendency to enter states of oscillatory synchrony [24]. It remains to be determined whether the decrease in gamma activity associated with cognitive deficits and the positive correlation between gamma activity and psychotic symptoms are due to different underlying mechanisms, manners of eliciting gamma, or cohorts of subjects. 

If, as suggested by multiple lines of evidence, altered neural synchrony underlies impairment of cognition in schizophrenia, then understanding the neural mechanisms normally involved in production of synchronized oscillations in neocortical circuits is crucial to develop new therapeutic interventions. Whereas several mechanisms have been proposed, production of rhythms via GABA-mediated inhibition is currently a leading candidate mechanism, as reviewed in the following.

## 3. Basic Mechanisms of GABA-Mediated Fast Synaptic Transmission

By definition, GABA neurons have the capacity to synthesize GABA from glutamate via the enzymatic activity of glutamic acid decarboxylase (GAD), for which there are two gene products of different molecular weight, GAD65 and GAD67 [25]. Whereas the GAD isoforms differ in a number of properties, their specific functional roles are not fully understood [25]. Interestingly, GAD65 and GAD67 are differentially expressed in GABAergic terminals in a cell type-specific manner (see below). 

**Figure 1:**
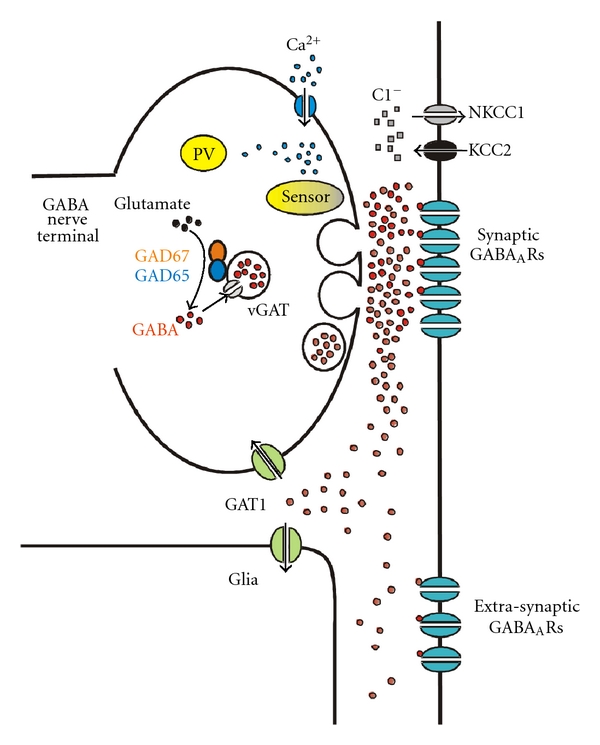
Scheme showing a nerve terminal from a parvalbumin- (PV-) positive GABA neuron shortly after an action potential triggered Ca^2+^-dependent GABA release, highlighting components currently hypothesized to be altered in schizophrenia. In PV terminals, GABA release is tightly synchronized with Ca^2+^ influx, possibly due to the proximity between voltage-dependent Ca^2+^ channels and release sites. PV is a relatively slow buffer that probably is unable to bind Ca^2+^ before activation of the Ca^2+^ sensor promotes vesicle fusion. Ca^2+^ buffering by PV mainly accelerates the decay of the intraterminal Ca^2+^ transient (see text). GAD65 and GAD67, possibly acting as a dimer, drive GABA synthesis in the cytosol near synaptic vesicles. Vesicles uptake newly synthesized GABA via the vesicular GABA transporter vGAT. Vesicle fusion rapidly and transiently raises GABA concentration in the synaptic cleft, briefly exposing post-synaptic GABA_A_ receptors (GABA_A_Rs) to a high concentration of GABA. As GABA escapes from the synaptic cleft after GABA_A_R activation, it may be taken up by the plasma membrane GABA transporter GAT1, apparently localized in the extrasynaptic neuronal membrane, as well as in glia. GAT1 therefore regulates the concentration of GABA reaching extrasynaptic GABA_A_Rs and synaptic GABA_A_Rs at other synapses (not shown in the scheme). The direction and magnitude of the chloride current produced by postsynaptic GABA_A_R activation is regulated by the transporters KCC2 and NKCC1, which uptake and extrude chloride, respectively, setting the equilibrium potential for the GABA_A_ current, *E*_GABA_A__. Since PV accelerates the decay of the intraterminal Ca^2+^ transients, a decrease of PV in schizophrenia may facilitate repetitive GABA release, such as that observed during gamma oscillation episodes. A decrease of GAD67 levels in schizophrenia would reduce the cytosolic GABA concentration near synaptic vesicles. Because vGAT levels appear to be unaffected in schizophrenia, reduced GAD67 may lead to lower intravesicular GABA concentration, therefore decreasing the peak GABA concentration in the synaptic cleft and weakening the postsynaptic response. In schizophrenia, at some synapses postsynaptic GABA_A_R density appears to be decreased, further weakening synaptic transmission, whereas at other synapses GABA_A_R density is increased, possibly due to compensatory receptor upregulation. In schizophrenia, KCC2 and NKCC1 mRNA levels are normal, but two kinases that strongly regulate KCC2 and NKCC1 may be altered in ways that render an *E*_GABA_A__ value more depolarizing than normal. Finally, reduced GAT1 in schizophrenia may alter the effects of synaptically released GABA via an exaggerated activation of extrasynaptic and heterosynaptic GABA_A_Rs. Alternatively, GAT1 activity may be reduced to compensate lower GABA levels due to GAD67 deficiency.

GAD-mediated GABA synthesis occurs in the cytosol, and GABA is transported into synaptic vesicles by the vesicular GABA transporter vGAT (Figure 1). Shortly after an action potential arrives at the nerve terminal, vesicular GABA release is triggered with a certain probability and in a Ca^2+^-dependent manner. In PV neuron terminals, PV may act as a Ca^2+^ buffer that binds residual Ca^2+^ after activation of the Ca^2+^ sensor that triggers vesicular GABA release. At all synaptic connections from cortical GABA neurons thus far studied, the effects of synaptically released GABA are mediated by GABA_A_ receptors (GABA_A_Rs), as the postsynaptic response is abolished by GABA_A_R antagonists in hippocampus [26–33] and neocortex [34–41]. In contrast, GABA_B_ receptors mediate the postsynaptic effects of GABA only at connections from GABA neurons of the neurogliaform cell subtype [42]. 

Postsynaptic GABA_A_Rs are heteropentamers composed of subunits from 7 different families (*α*_1–6_, *β*_1–3_, *γ*_1–3_, *δ*, *ε*, *θ*, and *ρ*_1–3_) typically combined following a 2*α* : 2*β* : *γ* stoichiometry to form a GABA-activated chloride channel [43, 44]. Importantly, the subunit composition of the GABA_A_R complex determines many of its functional properties. For instance, GABA-activated chloride currents produced by *α*1 subunit-containing GABA_A_Rs (*α*1-GABA_A_Rs) have much faster decay kinetics than currents mediated by GABA_A_Rs containing other *α* subunits [43]. 

The subunit composition also determines important pharmacological properties of the GABA_A_Rs. Benzodiazepines bind to GABA_A_Rs via a binding site localized at the interface between *α* and *γ* subunits [45, 46] and may modulate (potentiate or decrease) GABA_A_R function in an *α* subunit-selective manner [47]. For example, zolpidem enhances GABA effects preferentially at *α*1-GABA_A_Rs, whereas the *α*3IA and *α*5IA compounds are inverse agonists preferentially at *α*3-GABA_A_Rs and *α*5-GABA_A_Rs, respectively [47]. Other drugs, including TPA023 (also named MK0777), TPA023B, TPA123, and TPA003 have comparable binding affinity at *α*1-, *α2*-, *α*3-, and *α*5-GABA_A_Rs but may lack pharmacological effects at certain GABA_A_R subtypes. In particular, TPA023 is a partial agonist at *α2*- and *α*3-GABA_A_Rs but has no agonist efficacy at *α1*- and *α*5-GABA_A_Rs [48].

The magnitude and direction of the ionic current flowing through GABA_A_Rs depends on its driving force or difference between its electrochemical equilibrium potential (*E*_GABA_A__) and the resting membrane potential (*V*_mr_) in the plasma membrane compartment where GABA_A_Rs are located [49]. Because GABA_A_R channels are largely permeable to chloride, *E*_GABA_A__ is close to the chloride equilibrium potential (*E*_Cl_) and therefore *E*_GABA_A__ depends on the sodium-potassium-chloride cotransporter 1 (NKCC1) and the potassium-chloride co-transporter 2 (KCC2), which mediate chloride uptake and extrusion, respectively [43]. Importantly, since the active GABA_A_R conductance tends to “clamp” the membrane potential at *E*_GABA_A__ [49], if *E*_GABA_A__ is negative relative to *V*_mr_  (*V*_mr_ > *E*_GABA_A__), the GABA_A_R currents are hyperpolarizing, whereas if *E*_GABA_A__ is positive to *V*_mr_  (*E*_GABA_A__ > *V*_mr_), the GABA_A_R currents produce depolarization. Commonly, *E*_GABA_A__ is negative to *V*_mr_, however, in certain cell types or subcellular compartments (and in general, early in brain development) *E*_GABA_A__ > *V*_mr_ [43]. 

Whether the GABA_A_R conductance has inhibitory or excitatory effects depends on the relation between *E*GABA_A_ and the voltage threshold for action potential firing (*V*_th_), which is always depolarized relative to *V*_mr_  (*V*_th_ > *V*_mr_). When *V*_th_ > *V*_mr_ > *E*_GABA_A__, the chloride current is hyperpolarizing and clearly inhibitory because it shifts the membrane potential away from firing threshold, thus reducing the probability of firing. In contrast, if *E*_GABA_A__ > *V*_th_ > *V*_mr_, the chloride current is excitatory because it tends to depolarize the membrane above *V*_th_. However, if *V*_th_ > *E*_GABA_A__ > *V*_mr_, the GABA_A_R conductance is inhibitory because, even though *E*_GABA_A__ > *V*_mr_, shunting by the active GABA_A_R conductance keeps the membrane potential below firing threshold. Importantly, GABA_A_R-mediated inputs that depolarize the membrane below *V*_th_  (*V*_th_ > *E*_GABA_A__ > *V*_mr_) can have dual, time-dependent inhibitory/excitatory effects. Initially, when the GABA_A_R conductance is active, the net effect of the synaptic input is inhibitory because of the shunting effect [50]. However, the depolarizing postsynaptic potential outlasts the duration of the GABA_A_R conductance, thus increasing the firing probability once the GABA_A_R conductance decays [50]. Importantly, the depolarizing GABA_A_R-mediated post-synaptic potential may be amplified by voltage-dependent Na^+^ currents localized perisomatically, possibly in the initial segment of the axon [51, 52], enhancing its excitatory effect. The inhibitory versus excitatory effect of the GABA_A_R conductance may be dynamic, because *V*_mr_ and *V*_th_ are subject to modulation and change over time. In addition, *E*_GABA_A__ may vary between cell types and subcellular compartments, depending on the NKCC1/KCC2 activity ratio [43].

It has been commonly suggested that uptake of extracellular GABA by plasma membrane transporters could help terminate the synaptic effect of GABA and thus the duration of the inhibitory postsynaptic current (IPSC). In the CNS, GABA uptake is largely mediated by the plasma membrane GABA transporter 1 (GAT1) which translocates GABA through the neuronal and glial membrane (Figure 1). Interestingly, recent experiments indicate that GAT1 does not control the time course of GABA_A_R-mediated IPSCs, since the duration of IPSCs produced at single synapses is not affected by pharmacological inhibition of GAT1, nor in GAT1 knockout mice [53–56]. The finding that GAT1 does not control IPSC duration may be explained by GAT1's predominantly extrasynaptic localization [57–62] and by the slow GAT1-mediated GABA translocation rate [63, 64] compared with the rapid GABA_A_R activation by synaptic GABA [43]. Other experiments suggest that GAT1's main role is preventing intersynaptic GABA spillover [53, 56], (e.g., the unintended activation of GABA_A_Rs at a given synapse by GABA released at adjacent synapses). Four different GABA transporters have been cloned: GAT1, GAT2, GAT3, and BGT1 [65]; therefore, it is possible that some of the GABA transporters different from GAT1 have properties consistent with uptake-mediated control of IPSC duration. However, GAT2 is only found during very early brain development and BGT1 is not abundant in brain [65]. GAT3 is mostly localized in glia, and the effects of GAT3 blockade indicate that, similar to GAT1, GAT3's main role is reducing the effects of GABA spillover [66].

## 4. Mechanisms of GABA_**A**_R-Mediated-Gamma- Band Synchronization

**Figure 2:**
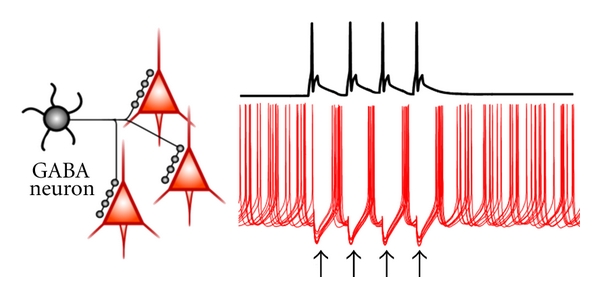
A model for GABA_A_R-mediated synchronization mechanisms. The left panel shows a group of pyramidal cells that are common postsynaptic targets of an inhibitory GABA neuron. Perisomatic-targeting GABA neurons such as that in the scheme produce stronger inhibition than GABA neuron subtypes that target the dendrites. The right panel shows, above (black trace), the membrane potential of the GABA neuron which remains at rest before and after firing a sequence of four action potentials. The red traces below show the membrane potential simultaneously recorded from the postsynaptic pyramidal neurons, which are firing in response to a continuous excitatory input. Note that, before the GABA neuron starts firing, the pyramidal cells fire in an asynchronous manner. Shortly after the first GABA neuron spike, an inhibitory postsynaptic potential (IPSP) is produced (first black arrow) which simultaneously inhibits the firing of all pyramidal neurons. After the IPSP-mediated inhibition decays, the pyramidal neurons fire in nearly synchrony. Note that a similar postinhibition synchronization is observed with each of the IPSPs evoked by the interneuron spikes (each IPSP is denoted by a black arrow). Once the GABA neuron stops firing, pyramidal cell activity rapidly becomes asynchronous. Also note that a single action potential in a GABA neuron would synchronize the pyramidal cells only once, whereas production of a synchronized oscillation requires rhythmic GABA neuron firing. An oscillation episode composed of four cycles is shown in the figure. The production of synchronized oscillations via this mechanism may be impaired by various alterations of GABA_A_R-mediated synaptic inhibition in schizophrenia (see Figure 1 and main text).

The mechanisms by which GABA_A_R-mediated inhibition may synchronize postsynaptic cell activity have been reviewed in detail previously [7, 8, 67, 68].  Figure 2 illustrates a group of pyramidal neurons firing asynchronously in response to some excitatory inputs that receive common GABA_A_R-mediated hyperpolarizing inhibition. If such GABA-mediated hyperpolarizing input is strong enough, then the postsynaptic neurons will be inhibited together during a certain time window and, as the GABA_A_R inhibitory effect decays, will escape from inhibition to resume firing nearly in synchrony (Figure 2). Such postinhibition synchronous spiking of pyramidal cells can be elicited by single-GABA neurons [28] and readily generates synchrony throughout large numbers of neurons in computational network models [7, 67]. Therefore, post-inhibition synchronous spiking is a strong candidate mechanism for production of neural synchrony. Alternative synchronization mechanisms, which are not reviewed here, involve gap junctions connecting pyramidal cell axons [8] or noisy but correlated inputs [69]. 

Importantly, neuronal synchrony is commonly observed during episodes of rhythmic/oscillatory network activity, especially in association with cognitive tasks [70, 71]. Therefore, the circuit mechanisms of synchronized oscillations via GABA_A_R-mediated inhibition must involve rhythmic interneuron firing and trains of IPSCs in their postsynaptic target cells. As multiple subclasses of GABA neurons exist [72], a crucial issue is whether specific subtypes are involved in the mechanisms of synchronized oscillations. Synchronized oscillations occur at different frequency bands [70], including theta (*∼*4–10 Hz), beta (*∼*15–30 Hz), and gamma (*∼*30–80 Hz). Whether oscillations of all frequency bands depend on GABA_A_R-mediated inhibition and, if so, on particular GABA neuron subtypes is still a matter of investigation [73]. Here we focus on models for the mechanisms of gamma oscillations, which are commonly induced during cognitive tasks and seem to be impaired in the cortex of patients with schizophrenia. 

**Figure 3:**
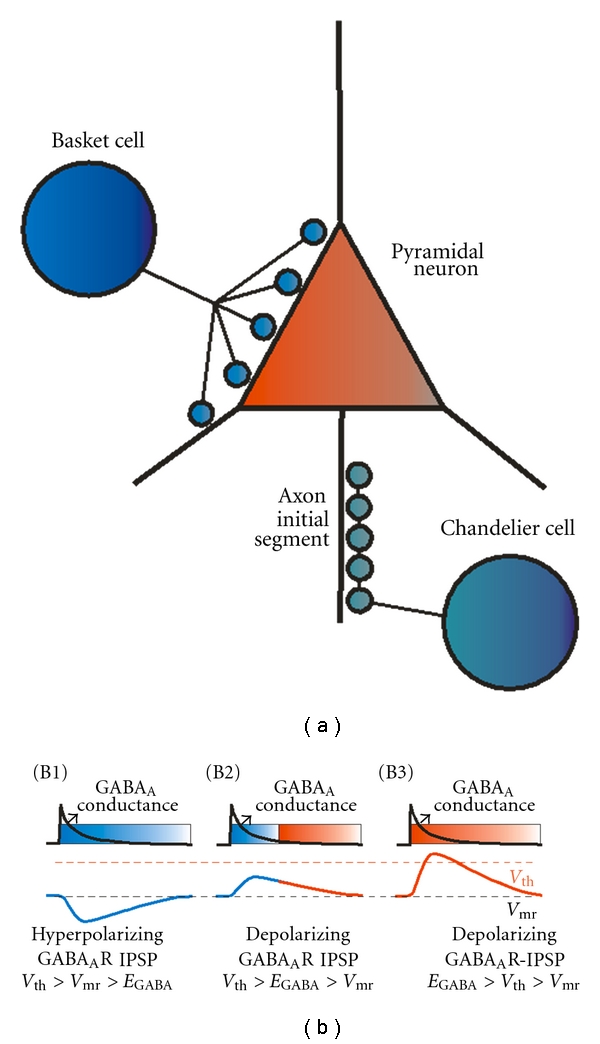
Diversity of perisomatic-targeting GABA neuron-mediated inhibition in cortical circuits may be due to differences in the reversal potential for the GABA_A_-mediated current. (a) The scheme illustrates targeting by basket cells (either parvalbumin- or cholechystokinin-positive), which contact the soma and the proximal/perisomatic dendritic compartments, and by chandelier or axoaxonic neurons, which contact the axon initial segment. (b) The schemes illustrate differences in the postsynaptic effect of a GABA_A_R conductance according to the value of the reversal potential for the GABA_A_ current (*E*_GABA_A__) relative to the resting membrane potential (*V*_mr_) and the threshold potential to fire spikes (*V*_th_). In (B1) to (B3), the time course of the GABA_A_R-mediated conductance, chosen to be identical in all panels, is shown by the black traces and the IPSPs are shown by the blue/red traces. Note that the IPSP time course is always slower than the GABA_A_ conductance, although in the scheme the difference in time course is somewhat exaggerated for illustration purposes and does not match the actual time scales. (B1) Illustration of cases in which *E*_GABA_A__ is negative relative to *V*_mr_  (*V*_th_ > *V*_mr_ > *E*_GABA_A__), and the GABA_A_ conductance generates a hyperpolarizing IPSP (all blue IPSP trace). As the IPSP outlasts the GABA_A_ conductance, the duration of the inhibitory effect of the synaptic input (shown by the shaded blue rectangle) is extended by the membrane hyperpolarization that remains after the GABA_A_ conductance decays. (B2) Illustration of cases in which the IPSP is depolarizing because *E*_GABA_A__ is positive relative to *V*_mr_  (*V*_th_ > *E*_GABA_A__ > *V*_mr_). Just as the hyperpolarizing IPSP, the depolarizing IPSP outlasts the GABA_A_ conductance; however, in this case *E*_GABA_A__ is below *V*_th_, and therefore the depolarizing IPSP could have a dual inhibitory/excitatory effect (blue/red IPSP trace), initially producing shunting inhibition which lasts approximately the same time as the GABA_A_ conductance (shaded blue/red rectangle), followed by an enhanced excitability of the postsynaptic neuron due to the remaining phase of the depolarizing IPSP. (B3) Illustration of cases in which *E*_GABA_A__ is positive relative to *V*_th_  (*E*_GABA_A__ > *V*_th_ > *V*_mr_). In this case, the depolarizing IPSP has a purely excitatory effect (shaded red rectangle). Basket cells are thought to produce hyperpolarizing GABA_A_R-mediated inhibition of pyramidal cells such as that illustrated in (B1). In contrast, the effect of chandelier neuron inputs is currently debatable, some studies suggesting an excitatory as that in (B3), other studies suggesting a purely inhibitory effect. Here, we suggest that depolarizing chandelier cell inputs may have a dual inhibitory/excitatory effect, illustrated in (B2), which could synchronize postsynaptic cells as described in Figure 2, although the depolarizing nature of the IPSP may accelerate the timing of synchronous firing after the postsynaptic cells escape from inhibition.

Synchronization by the GABA_A_R-mediated mechanism described in Figure 2 requires sufficiently strong GABA synapses activating a relatively large GABA_A_ conductance via inhibitory inputs localized near the site of action potential initiation. In pyramidal cells, action potentials are commonly triggered near the axon initial segment (AIS), the axonal compartment that is closest to the soma [74]. Therefore, inhibitory inputs onto the perisomatic membrane compartment (soma, proximal dendrites, and AIS) produce stronger inhibition than inputs onto distal dendrites [75, 76], suggesting that perisomatic-targeting GABA neurons may be crucially involved in production of synchronized oscillations. Three main subtypes of perisomatic-targeting GABA neurons exist in neocortex and hippocampus, namely, the parvalbumin-positive and the cholecystokinin-positive basket cells (PVBCs and CCKBCs) and the PV-positive chandelier cells (PVChCs). Both PVBCs and CCKBCs innervate pyramidal cells at the soma and proximal dendrites (Figure 3), whereas PVChCs synapse exclusively onto the AIS (Figure 3). Because a synaptic GABA_A_R conductance has stronger inhibitory effect the closest it is localized to the site of spike initiation [50], PVChC inputs onto the AIS are predicted to have the strongest inhibitory power. 

Surprisingly, some recent studies suggested that synaptic input from PVChCs is actually excitatory, since stimulation of PVChCs frequently initiates spikes in postsynaptic pyramidal cells via GABA_A_R activation [40]. In addition, electron microscopy studies support the idea that PVChC inputs are excitatory, as they show very low levels of KCC2 in the AIS compared to the soma or dendrites [40, 77]. Since KCC2 extrudes chloride, lower AIS levels of KCC2 should produce a more positive *E*_GABA_A__, resulting in a depolarizing GABA_A_R current (Figure 3) [43]. In fact, in paired recordings using experimental conditions that preserve the physiological intracellular chloride concentration, PVChC inputs depolarize the postsynaptic pyramidal neurons [40, 78], whereas in identical experimental conditions PVBC inputs are hyperpolarizing [40, 78], consistent with higher levels of KCC2 transporters in the somatic membrane [40, 77]. Experiments with uncaging of GABA onto GABA_A_Rs in specific membrane compartments similarly showed a more depolarized *E*_GABA_A__ at the AIS compared with the soma and dendrites [79]. 

An excitatory depolarizing GABA_A_R-mediated input by PVChCs is inconsistent with their participation in inhibition-based synchronization. However, although *E*_GABA_A__ at the AIS is 10–20 mV depolarized before *V*_mr_, it may be negative relative to *V*_th_ [40, 78, 79]. As highlighted above, when *V*_th_ > *E*_GABA_A__ > *V*_mr_, the GABA_A_ conductance has a dual inhibitory/excitatory effect (Figure 3), which in the case of PVChC inputs may be amplified by their proximity to the spike initiation site. Such inhibitory/excitatory effect may contribute to synchronization of postsynaptic cell activity at gamma band frequency [7, 80]. Specifically, since the postsynaptic potential outlasts the duration of the GABA_A_R conductance (Figure 3), once the conductance is deactivated, the depolarizing postsynaptic potential accelerates the post-inhibition synchronous spiking, facilitating synchronization at gamma frequency. Such a mechanism operates at GABA_A_R-mediated synapses onto GABA neurons [7, 80] and could also apply at PVChC inputs onto pyramidal neurons. 

The depolarizing effect of PVChCs described in neocortex [40, 78] contrasts with pioneer paired recording experiments showing that either PVBC or PVChC inputs hyperpolarize hippocampal pyramidal neurons [26]. A hyperpolarizing effect of hippocampal PVBCs and PVChCs was also suggested recently by experiments using conditions that preserve the intracellular chloride concentrations [81], but that are different from the intracellular chloride-preserving conditions employed in studies of neocortical PVChC inputs [40, 78]. Interestingly, the AIS membrane has low levels of KCC2 in neocortical [40] and hippocampal [77] pyramidal cells. The discrepancies between findings for hippocampal versus neocortical PVChCs highlight the need for further research to clarify the enigmatic role of this class of GABA neurons [82]. Whether excitatory or inhibitory, the unique properties of PVChC inputs suggest that the reported alterations of these cells must significantly contribute to cortical circuit dysfunction in schizophrenia [83]. 

In contrast to PVChCs, both PVBC and CCKBC inputs are hyperpolarizing (*V*_mr_ > *E*_GABA_A__), although *E*_GABA_A__ is slightly, but significantly, different for PVBC versus CCKBC inputs [84]. Such differences in *E*_GABA_A__ were attributed to the activity of the voltage- and chloride-dependent chloride channel ClC-2, which helps maintaining a low internal chloride concentration (thus a hyperpolarized *E*_GABA_A__) at inputs from PVBCs but not from CCKBCs [84]. The ClC-2-dependent regulation of internal chloride at PVBC inputs is dependent on the extent of GABA_A_R activation [84]. These findings highlight a degree of functional diversity between BC subtypes, showing that PVBC inputs, but not CCKBC inputs, possess a mechanism to prevent internal chloride accumulation during high-frequency neuronal activity such as that observed in GABA neurons participating in gamma oscillations. 

**Figure 4:**
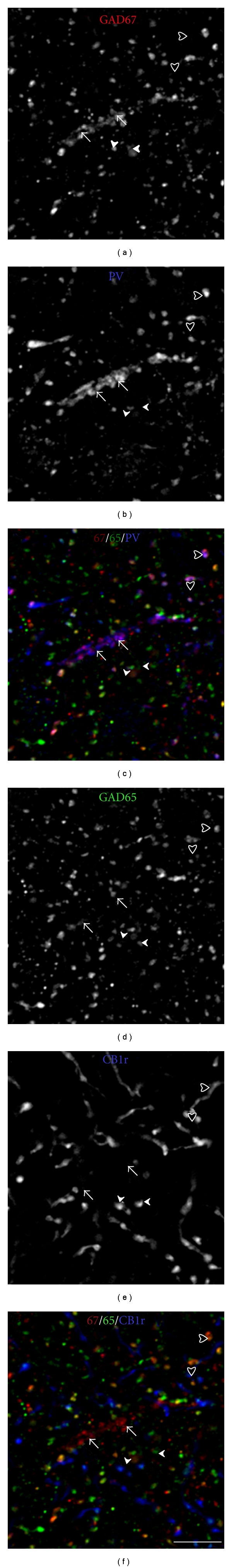
Relative levels of GAD65 and GAD67 in PVCh, PVBC, and CCKBC terminals. Cryostat sections of monkey PFC tissue (40 *μ*m thick) were quadruple labeled for GAD65, GAD67, PV, and cannabinoid receptor 1 (CB1r). Among inhibitory synaptic boutons, CB1r is exclusively expressed in those CCK-positive neurons and is completely absent in terminals of PV-positive neurons. Presynaptic CB1rs are also present in excitatory synapses; however, the antibody used in the studies illustrated in this figure exclusively reveal CB1r expression in inhibitory boutons (see main text for details). Therefore, CB1r expression is a marker of CCK cell terminals. Single channel (a)-(b) and (d)-(e) and merged (c) and (f) projection images of deconvolved image stacks (2 z-planes 0.25 *μ*m apart). Since four labels cannot be displayed together in a single image, they have been separated into two RGB images that contain GAD67 (red), GAD65 (green), and PV (blue (c)) or CB1r (blue (f)). Arrows: PV cartridge; solid arrowheads: CB1r^+^/GAD65^+^ and GAD67^−^ terminals; open arrowheads: GAD65^+^/GAD67^+^/PV^+^ terminals. Bar = 10 *μ*m.

Functional diversity between PVBCs, PVChCs and CCKBCs is also suggested by recent data demonstrating that the relative levels of GAD65 and GAD67 proteins in nerve terminals of these cells vary significantly in a cell-type-specific manner [85]. Using immunocytochemical labeling combined with a quantitative fluorescence microscopy methodology [86], the colocalization of GAD65 and GAD67 proteins in the same terminals was assessed for PVBCs, PVChCs and CCKBCs (Figure 4). The latter cells were identified using an antibody that detects the cannabinoid 1 receptors expressed by inhibitory terminals. Importantly, in the prefrontal cortex nearly all cannabinoid 1 receptor-expressing cells detected using this antibody are immunoreactive for CCK [87]. This assessment showed that the ratio of GAD67/GAD65 expression varied significantly across each type of terminal, with very high (15.42) and very low (0.18) ratios observed in terminals of PVChCs and CCKBCs, respectively, and a ratio of 1.49 for PVBC terminals [85]. These data reinforce the idea that synaptic connections from different perisomatic-targeting GABA neuron subtypes are functionally diverse. The impact of different GAD67/GAD65 ratio on the properties of the GABA synapses studied remains enigmatic because the differential functional roles of GAD67 and GAD65 are still poorly understood. In mice, GAD67 deficiency produces major alterations, as GAD67^−/−^ animals are born with cleft palate (which is lethal) and with *∼*90% reduction of bulk GABA concentration in brain tissue [88, 89]. In contrast, GAD65^−/−^ mice survive into adulthood, displaying *∼*20% reduction in total brain GABA concentration [90] together with increased susceptibility to seizures [90, 91], increased anxiety [92], and altered fear conditioning [93]. In synapses of GAD65^−/−^ mice, GABA_A_R-mediated synaptic transmission appears normal during low-frequency stimulation but is strongly impaired at stimulation frequency of 30 Hz or higher [94, 95]. In mice with cerebellum-specific GAD67 deficiency [96], GABA_A_R-mediated transmission is markedly weaker even at low stimulus frequency [96], suggesting a crucial role of GAD67 in baseline synaptic transmission. Whether the effects of GAD67 deficiency on cerebellar synapses are observed in cortical synapses as well remains to be determined. Importantly, GAD67 deficiency markedly impairs the maturation of cortical GABAergic synaptic connections [97]. Therefore, testing the role of GAD67 in synaptic transmission independent of its role in synapse development requires a paradigm in which GAD67 is decreased once GABA synapse maturation has been completed. In addition, GAD65 and GAD67 both comprise *∼*50% of total GAD protein in mouse cortex, while GAD65 comprises *∼*70% in the rat cortex [98]. The GAD65 and GAD67 proportions of the total GAD protein in human cortex is unknown; however, the differences between mouse and rat strongly stress the need to study GAD function in molecularly relevant systems when trying to understand their roles in human disease. 

GABA_A_R-mediated inputs involved in gamma band synchronization must inhibit their postsynaptic neurons for a time compatible with the gamma oscillation period. For instance, long-lasting postsynaptic currents such as those activated by GABA_B_ receptors (which may last hundreds of milliseconds) are inconsistent with gamma synchrony, as postsynaptic neuron inhibition would be longer than the typical gamma cycle period. Whereas all GABA_A_R subtypes produce faster currents than GABA_B_ receptors, the GABA_A _current duration depends on the subunit composition [43, 99]. As mentioned, recombinant *α*1-GABA_A_Rs produce currents with the fastest decay [100]. Therefore, PVBCs may produce IPSCs with faster decay, as *α*1-GABA_A_Rs predominate at inputs from PVBCs [33, 41, 101, 102] whereas *α*2-GABA_A_Rs predominate at both PVChC and CCKBC inputs [41, 101–103]. Interestingly, PVBCs, CCKBCs, and PVChCs elicit in pyramidal cells uIPSCs with nearly identical kinetics [39, 104–107]. Therefore, in addition to the GABA_A_R subunit composition, the IPSC kinetics are determined by additional factors that may not affect recombinant GABA_A_Rs. Such factors include GABA_A_R subunit phosphorylation and other posttranslational modifications [108, 109], and the time course of the GABA concentration transient to which GABA_A_Rs are exposed [41]. 

Although PVBCs and CCKBCs produce IPSCs with similar duration during individual GABA release events, CCKBCs distinctively produce multiple asynchronous release events after single-action potentials [104]. Multiple GABA release events may prolong the postsynaptic inhibitory effect of each CCKBC spike [104]. In contrast, PVBC terminals produce a single synchronous GABA release event per presynaptic action potential [104, 110]. Asynchronous release from CCKBC terminals has been observed in multiple studies [104, 107, 111–115] suggesting that it is a fundamental property that prolongs the inhibitory effect of CCKBCs on postsynaptic neurons possibly linking their activity with synchronization at frequency bands lower than gamma. 

The data reviewed above suggest that, relative to CCKBCs and PVChCs, PVBCs have unique properties consistent with a crucial role in the mechanisms of gamma band synchrony. However, such data do not directly assess involvement of any of these GABA neuron subtypes in the gamma oscillation mechanisms. Interestingly, some electrophysiological studies more directly indicate that among perisomatic-targeting GABA neuron subtypes, PVBCs are most likely to be involved in the production of gamma oscillations. For instance, whereas both BCs and ChCs are active during gamma oscillations in vivo [116–118] and in vitro [119–124], the firing of CCKBCs and PVChCs is more weakly coupled with the gamma oscillation cycle than PVBC firing [124], although CCKBC and PVChC firing is strongly coupled with theta oscillations [125–127]. Furthermore, gamma oscillations are significantly reduced or abolished by suppressing PV cell activity with optogenetic methods that do not directly affect CCKBCs [128] or by stimulation of presynaptic opioid receptors that suppress GABA release from PVBCs but not from CCKBCs or ChCs [124]. Therefore, perisomatic GABA_A_R-mediated currents from PVBCs appear to be the main source of GABA_A_R-mediated synchronization in the gamma frequency band. 

Essential for modeling the circuit mechanisms of gamma synchronization is to understand how PVBCs are normally recruited to fire rhythmically at gamma frequency. Recruitment of PVBC firing depends on activation of not only excitatory but also inhibitory synaptic inputs onto them since PVBCs target other GABA neurons, including nearby PVBCs [129, 130], and are also inhibited by inputs from different classes of GABA neurons, including the CCKBCs [112]. Gamma oscillations are successfully generated in computational model networks that rely on reciprocal inhibition between GABA neurons and are thus called ING, for Interneuron Network Gamma [131–133]. In ING models, GABA neurons are recruited by a strong tonic excitation that drives them to fire at a frequency above gamma, and the reciprocal inhibition synchronizes GABA neuron firing at a frequency inversely related to the IPSC duration, falling within gamma range for durations typical of *α*1-GABA_A_R-mediated IPSCs [132, 133]. Whereas gamma rhythms possibly induced by ING-like mechanisms have been observed experimentally [134], the actual source of the strong tonic excitation onto GABA neurons required by ING models is unclear. Metabotropic glutamate receptors [67, 134] or kainate receptors [135] could provide such a tonic drive, although this possibility is supported, only indirectly, by findings obtained with AMPA- and NMDA-mediated synaptic transmission blocked. A recent study employing genetically engineered mice with a deletion of GABA_A_R expression in PVBCs [136] directly tested whether inhibition onto PVBCs is necessary to generate gamma oscillations, as predicted by the ING models. In such mice, GABA_A_R-mediated IPSCs were abolished exclusively in PVBCs [136] and theta oscillations and their coupling with gamma oscillations were severely disrupted [136]. However, in such mice hippocampal gamma oscillations in vivo were intact as compared with wild-type mice [136]. These data argue against the ING model for gamma band synchrony and suggest that inhibition onto PVBCs, potentially from CCKBCs [112], is crucial for coupling theta and gamma oscillations. Such theta-gamma coupling is thought to be important for cognitive function [137]. 

As the role of ING mechanisms in gamma oscillation production continues to be tested, some studies favor an alternative model, known as Pyramidal Interneuron Network Gamma (PING), which depends on recurrent excitatory-inhibitory synaptic interactions. In PING, PVBCs are recruited by phasic glutamate-mediated inputs from the pyramidal cells, and the PVBCs provide strong feedback inhibition that synchronizes pyramidal cell firing [67, 121, 138]. The PING model predicts that during the gamma oscillation cycle PVBCs fire after the pyramidal neurons, with timing consistent with monosynaptic recruitment by pyramidal cells [138]. The spike timing of pyramidal cells and putative BCs during gamma oscillations in awake behaving animals is actually consistent with the PING model, as BCs fire 2-3 ms later than pyramidal neurons [139, 140]. Similar findings were obtained for pyramidal and PVBC spikes during gamma oscillations in hippocampal and neocortical brain slices [119, 120, 122, 123, 141]. PING models also predict the presence of trains of gamma frequency IPSCs in pyramidal neurons and trains of gamma frequency EPSCs in PVBCs [67]. Evidence consistent with IPSC trains in pyramidal cells was obtained for gamma oscillations in vivo [117, 142] and in vitro [119–123, 141, 142]. In addition, during gamma oscillations in vitro, PVBCs display rhythmic EPSCs highly synchronized with the gamma rhythm [120–122]. Interestingly, optogenetics experiments show that driving PV neurons by nonrhythmic excitatory inputs is sufficient to generate gamma synchrony via feedback inhibition [128], a finding also consistent with the PING model of gamma.

PING mechanisms rely on recruitment of PVBCs by phasic excitatory input [138]; therefore, the properties of glutamate synapses onto PV neurons are extremely relevant for models of gamma oscillations. Interestingly, schizophrenia has been hypothesized to be associated with a deficit of glutamate transmission [143], more specifically with hypofunction of NMDA receptors, particularly in GABA neurons [143]. Moreover, some studies have suggested that NMDA hypofunction could especially affect PV cells [144–146]. Therefore, an important question is the following: what are the subtypes of glutamate receptors that mediate synaptic activation of PV GABA neurons? The answer to this question is relevant in the context of alterations of gamma synchrony in schizophrenia, because if NMDA receptors are important to recruit PV neurons in a PING mechanism of gamma, then NMDA hypofunction could be linked to deficits of gamma synchrony in schizophrenia. Data from recent studies showed that systemic administration of NMDA receptor antagonists increase the firing rate of putative pyramidal neurons and decrease the firing of putative inhibitory cells in the PFC in vivo [147], suggesting that NMDA receptors may be crucial to recruit inhibition. An important question not directly addressed by such studies [147] is whether the inhibitory neurons dependent on NMDA receptors belong to the PV-positive class of GABA neurons. Whereas several studies demonstrated that synaptic excitation of PV neurons is relatively NMDA receptor independent, until recently no studies directly compared the importance of NMDA receptors in synaptically evoked recruitment of PVBCs versus pyramidal neurons in neocortical circuits. Recent data from recordings in mouse PFC show that, compared with pyramidal cells, glutamate synapses onto PVBCs have EPSCs with faster decay and weaker NMDA receptor contribution [148], supporting the idea that the rapid activation of PVBCs [149] is largely dependent on fast AMPA receptor-mediated excitation. Moreover, in a computational model producing gamma oscillations via PING mechanisms, fast AMPA-mediated excitation of PVBCs was critical for gamma band synchronization because the slower decay time course of NMDA-mediated EPSCs disrupted gamma band synchrony [148]. Some studies indeed showed that gamma oscillations are not affected or are enhanced by NMDA receptor antagonists, whereas AMPA receptor antagonism completely abolished them [150–153]. Similarly, in mice with AMPA receptor deletion genetically engineered to occur exclusively in PV-positive neurons, gamma oscillations are strongly reduced [154]; however, NMDA receptor deletion selectively in PV-positive cells does not decrease and in fact increases gamma oscillation power [155]. The results of recent studies therefore suggest that, in mature cortical circuits, NMDA receptors only play a minor role in synaptically evoked excitation of PV-positive neurons and therefore on gamma oscillations produced via PING mechanisms. If indeed PV neuron excitation is normally weakly dependent on NMDA receptors, then such data suggest that excitatory synapses onto PV cells are an unlikely target of NMDA receptor hypofunction mechanisms in schizophrenia. Importantly, although in most cases gamma synchrony is unaffected by NMDA receptor blockade, the effects of NMDA receptor antagonists on gamma oscillations may vary with cortical region or layer, in some cases ketamine producing a decrease, in others producing an increase in gamma power [152, 156]. In addition, some data show that whereas mature PVBCs display a relatively small NMDA receptor mediated component in synaptic responses [148, 157, 158], such NMDA component is substantially more prominent in immature PVBCs [157, 158]. Such findings suggest the interesting possibility that alterations of NMDA receptor-signaling during early brain development could alter PVBC function in ways that persist into adulthood, thus changing the role of PVBCs in mature local circuits. Interestingly, a recent study showed that a genetically engineered deletion of NMDA receptors from PV-positive cells does not have significant effects if the deletion is produced in the brain of adult mice [159]. However, a similar deletion produced during early brain development caused behavioral alterations in adult mice, some of which resemble behavioral dysfunction in patients with schizophrenia [159].

## 5. Postnatal Development of GABA-Mediated Synaptic Inhibition

Schizophrenia is hypothesized to be a neurodevelopmental disorder based on data linking the disease with adverse events during pre- and perinatal periods and the presence of cognitive and behavioral deficits in childhood many years prior to the onset of psychosis during late adolescence and early adulthood [160]. Adolescence, the developmental transition from parent-dependent childhood to independent adulthood, is associated with significant changes in behavior and with marked improvements in cognitive control [161]. Moreover, gamma band synchrony emerges during childhood and continues to mature until early adulthood [162, 163]. The postnatal developmental trajectory of GABA-synaptic function may therefore suggest critical periods of vulnerability during which the mechanisms producing neural synchrony could become dysfunctional in schizophrenia. In what follows we review developmental studies of the functional properties of PVBCs and their synaptic connections in rodents and of GABA-related gene products studied in the human and nonhuman primate brain. 

**Figure 5:**
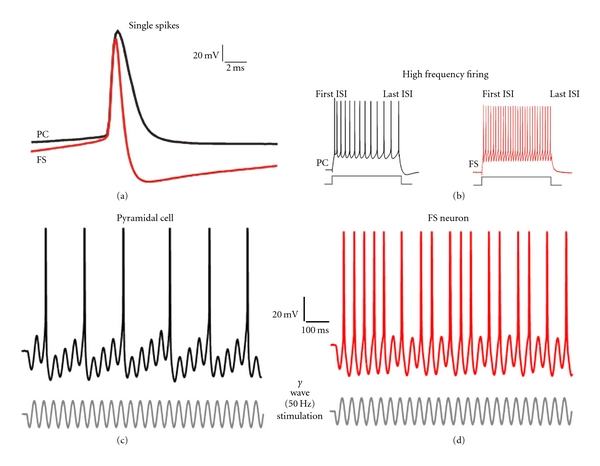
Intrinsic electrical properties of parvalbumin-positive GABA neurons. (a) Single action potentials in fast-spiking PV-positive neurons (FS) have significantly faster duration than pyramidal cell (PC) spikes or spikes in many other GABA neuron subtypes (not shown). (b) In response to sustained depolarizing current injection (500 ms rectangular current pulses shown below the traces), PCs produce high-frequency firing with significant spike-frequency adaptation as revealed by comparing the first and last interspike intervals (ISI). In contrast, adaptation of FS cell firing is much less significant or absent. (c) In response to gamma wave stimulation (sinusoidal current pulses shown below the traces), PCs show low capacity to respond with firing during each cycle of the gamma wave stimulus. (d) In response to gamma wave stimulation, FS cells show increased firing capacity, initiating spikes in the majority of gamma wave cycles. This property of the FS neuron membrane may contribute to the activation of FS neurons during gamma oscillations in vivo and is likely due to the resonance or frequency-preference properties (see text) that distinguish FS cells from PCs and also from other GABA neuron subtypes.

Mature PV neurons have a unique fast-spiking (FS) firing pattern (Figure 5), which includes very narrow action potentials and high frequency firing without the spike-frequency adaptation typically observed in pyramidal cells and other GABA neuron subtypes [72]. Although the nonadapting properties of FS cells are revealed with artificial stimuli (rectangular currents steps lasting several hundred ms), they correlate strongly with the particular ability of FS cells to respond to oscillatory inputs at gamma frequency (Figure 5), which is likely due to the gamma frequency resonance of the FS cell membrane [164]. Immature FS neurons, in contrast, have significantly slower action potentials and stronger spike-frequency adaptation, fail to respond efficiently to gamma frequency oscillatory inputs, and show much slower conduction velocity of action potentials along their axon [33, 165–167]. In rodent hippocampus, as well as in auditory, somatosensory, and prefrontal cortices, maturation of FS neuron electrical properties is complete by postnatal day 25 (P25) after which FS neurons display adult-like electrical properties [33, 157, 165–167]. 

Functional properties of the inhibitory connections onto excitatory neurons also differ markedly between developing and mature PVBCs: unitary IPSCs (uIPSCs), from immature PVBCs, are weaker and have slower decay time than uIPSCs produced by mature PVBCs [33, 168]. Such acceleration of uIPSC decay is explained by an increase in the contribution of *α*1-GABA_A_Rs with maturation [33]. A GABA_A_R subunit switch may also contribute to the developmental acceleration of the uIPSP decay [167] although developmental changes in the pyramidal cell membrane time constant contribute as well [167]. Mature FSBCs produce highly synchronous GABA release, in contrast to asynchronous release from mature CCKBCs [33, 104, 110]. However, GABA release is less synchronous and less reliable in synapses from immature FSBCs [33]. Postnatal maturation of the FSBC connections takes place relatively rapidly, as uIPSCs acquire mature properties by P28 [33, 167]. 

Inhibition onto FS cells also undergoes significant developmental changes. For example, uIPSCs at FSBC-to-FSBC connections mature within 3-4 weeks postnatally [33], whereas miniature IPSCs (mIPSCs, which represent GABA release at single synapses) recorded from FSBCs acquire adult-like properties by P25 [165]. Unitary EPSPs (uEPSPs) at immature pyramidal cell-to-FSBC connections have slow decay time, which accelerates markedly during development, reaching adult-like values by *∼*P22 [167]. Similarly, miniature EPSCs (mEPSCs) recorded from immature FSBCs are slow and show a developmental acceleration of their decay to reach very fast mEPSC decay values in mature FS cells [148, 157, 158, 165]. Fast-decaying EPSCs and EPSPs in mature FSBCs are largely mediated by AMPA receptors [148, 157, 158, 167, 169], suggesting that the rapid EPSC decay in FSBCs is due, at least in part, to a weak contribution from the slow-decaying NMDA currents [148, 157]. Actually, the developmental acceleration of EPSC decay is accompanied by a marked decrease in the contribution of NMDA currents which is still ongoing at *∼*P40 to *∼*P96 [157], which in rodents corresponds to the transition from adolescence to adulthood [170]. In contrast to PFC, in hippocampus, auditory, and somatosensory cortices, NMDA receptor contribution in FSBCs decreases prior to adolescence [167, 169, 171, 172].

Although the mechanisms controlling postnatal development of PVBC firing properties and their synaptic inputs and outputs are important candidates as the substrate of alterations in schizophrenia, they are still poorly understood. Neuregulin-1 is a trophic factor crucial for brain development that is encoded by a schizophrenia susceptibility gene and is highly expressed during late developmental periods and in adulthood [173, 174]. Among various neuregulin-1 receptors, the ErbB4 receptor, whose gene also confers schizophrenia susceptibility [173, 174], is enriched in GABA neurons, particularly in PV-positive cells [175, 176] where it facilitates GABA release [176], possibly mediating neuregulin-1 enhancement of gamma oscillations [177]. Neuregulin-1 signaling appears to regulate early development of GABA synapses [178] and, via ErbB4 receptors, may control development of PV neuron synapses [179]. Interestingly, ErbB4-mediated neuregulin-1 effects are crucial for development of excitatory synapses onto PVBCs [179, 180]. 

In parallel to the maturation of their firing pattern and synaptic connections, PVBCs undergo significant developmental morphological changes. For instance, the total length of the dendritic and axonal trees of PVBCs increases significantly from P6 to P25 [33], the number of axonal branch points increasing five times during this developmental period [33]. The number of postsynaptic neurons innervated by individual PVBCs also increases markedly with postnatal development [97], resulting in a higher functional connectivity between mature PVBCs and excitatory neurons [33]. A crucial factor regulating development of innervation patterns by PVBCs is GAD67-mediated GABA synthesis [97]. For instance, GAD67 knockdown in single PVBCs dramatically decreases formation of axon branches and synapses, as well as the number of postsynaptic neurons innervated by each PVBC [97]. Such effects of GAD67 knock-down are observed in organotypic cell cultures and in the primary visual cortex in vivo with GAD67 knock-down induced at P13 and the patterns of innervation examined at P20 or P32 [97]. The role of GAD67-mediated GABA synthesis in formation and/or stability of PVBC synapses may involve neuroligin-neurexin interactions and modulation of GABA receptor trafficking [181]. 

Because detailed molecular and biophysical analysis of developmental changes in GABA neuron and GABA synapse function is feasible only using animal models, especially rodents, an important question is how developmental time scales translate from animal to human brain [182]. Proper translation would require understanding if similar developmental stages are found in rodent and human brains, whether such developmental stages involve similar processes and underlying mechanisms, and whether developmental periods cover similar fractions of the total lifespan. Some developmental changes, for instance, excitatory synapse pruning, have similar proportional duration, with only the extent of synaptic pruning differing across mammalian species [183]. Also, functional maturation of glutamate synapses onto pyramidal cells occurs prior to adolescence in nonhuman primates [184], and in rodents [185–191].

Whereas some unique studies have assessed functional properties of PVBCs and PVChCs in the cortex of adult humans [40, 192, 193] and nonhuman primates [39, 184, 194–197], we lack information on the developmental trajectory of synaptic inhibition from primate PV neurons. Interestingly, functional properties of yet unidentified GABA synapses onto primate pyramidal neurons change during development through adolescence in a manner consistent with changes in the expression of gene products involved in GABA-mediated transmission [198]. Specifically, the decay time of GABA_A_R-mediated synaptic potentials accelerates during adolescence in parallel to changes in the protein and/or mRNA levels for *α*1 and *α*2 GABA_A_R subunits that would predict such acceleration [198].

The synaptic connections from PVChCs onto the pyramidal cell AIS form vertical arrays of multiple synaptic boutons that are usually easy to distinguish and typically called cartridges. Developmental properties of inputs from PVChCs onto the AIS can be studied using immunocytochemistry to detect biochemical markers that are concentrated at the cartridges in the AIS. In monkey PFC, the density of chandelier neuron axon cartridges immunoreactive for either PV or GAT1 changes markedly during postnatal development [199]. Although the precise time course differs for the two markers, the density of labeled cartridges is low in the newborn, increases to reach a peak prior to the onset of puberty, and then declines markedly to adult levels. Because cartridges are readily visualized with the Golgi technique over this same time period [200], the changes in PV- and GAT1-immunoreactive cartridges may reflect developmental shifts in the concentration of these proteins, rather than in the number of axon terminals, but this remains to be experimentally assessed.

Substantial developmental changes also occur postsynaptically at the AIS. In the adult cortex, the majority of *α*2-GABA_A_Rs are found in pyramidal cell AIS [201]. The detectability of GABA_A_ *α*2 subunits in AIS is very high in the early postnatal period and then steadily declines through adolescence into adulthood [199]. Immunoreactivity for ankyrin-G, *β*IV spectrin, and gephyrin (a scaffolding protein that regulates the clustering of GABA_A_Rs containing *α*2 subunits at AIS) [202–204] also exhibit substantial changes during postnatal development [205]. The densities of ankyrin-G and *β*IV spectrin immunoreactive AISs are greatest at birth and then sharply decline to reach relatively stable values by one year of age. In contrast, the relative density of gephyrin-immunoreactive AIS did not appear to change through the two postnatal years but then sharply decline through adolescence and into adulthood. 

The high density of AIS with detectable levels of ankyrin-G immunoreactivity in the first three postnatal months may reflect the recruitment to this location of a portion of the large number of GABA synapses that are formed in the monkey DLPFC during this developmental epoch [206]. Binding to ankyrin-G is also essential for the localization of many other membrane proteins to the AIS [207], including the voltage-gated Na^+^ channels that are required for action potential generation [208]. Thus, the high levels of ankyrin-G immunoreactivity may also indicate an increased capacity of pyramidal neurons for repetitive firing that parallels their increase in excitatory inputs during early postnatal development [206, 209]. The parallel relative densities of ankyrin-G-IR and *β*IV spectrin-SD-IR AIS likely reflect that ankyrin-G is required for the recruitment of *β*IV spectrin to AIS [210]. Although *β*IV spectrin is not essential for the formation of the AIS, it does appear necessary for maintenance of membrane structure and molecular organization [211], and thus the stability [210], of the AIS. Given the general role of spectrins in maintaining membrane integrity and elasticity [207], high levels of *β*IV spectrin during early postnatal development might insure the stability of AIS structure while PFC thickness is increasing [212]. 

Interestingly, in human PFC the levels of GAD67 mRNA increase progressively during prenatal and postnatal development through childhood until around the peripubertal period, followed by a plateau or mild decline during aging [213]. A similar pattern was reported for GAD67 mRNA expression during mouse [213] and monkey [214] cortical development, suggesting a highly conserved developmental trajectory of GAD67 expression across mammals. Studying protein expression by immunoblotting, a recent study found that GAD67 protein levels did not change across the lifespan in human visual cortex [215]. In contrast, GAD65 showed a progressive 60% increase until teenage years and young adulthood, followed by slight decline in older adults [215]. Two other presynaptic proteins involved in GABA transmission, the cannabinoid receptor 1 and vGAT, showed higher levels in infants and young children, which declined to adult-like levels in preteenage years [215]. The levels of PV mRNA increase markedly in postnatal human PFC, from very low perinatal levels until adult-like levels are reached by 2–5 years of age [216], an early developmental trajectory which is similar to that reported for PV mRNA and protein in rodent neocortex [217, 218]. 

Interestingly, a comparative analysis using immunolocalization of the chloride transporters NKCC1 and KCC2 revealed a very similar developmental trajectory in rat and human cortex [219]. NKCC1 levels peaking during perinatal development and decaying rapidly thereafter reach adult-like levels during childhood; conversely, KCC2 is undetectable perinatally and increases until reaching adult levels during childhood [219]. Since the NKCC1/KCC2 activity ratio determines whether GABA_A_R-mediated IPSCs depolarize or hyperpolarize the postsynaptic membrane, these data suggest that the very early developmental switch from excitatory to inhibitory effects at most GABA synapses [220] is highly conserved between rodent and human neocortex. 

Analysis of GABA_A_R subunit proteins during postnatal development in human visual cortex showed that *α*1 GABA_A_R subunit levels increase from <1 years until reaching adult levels at 13.5 years of age, whereas *α*2 GABA_A_R subunits decreased significantly with age to reach adult levels by ~10 years and *α*3 GABA_A_R subunit levels do not change significantly with age [215]. Consequently, the *α*1/*α*2 subunit protein ratio increased markedly with development attaining adult-like ratios at 4.5 years of age [215]. Remarkably, very similar developmental trajectories were found for the levels of GABA_A_R subunit mRNAs in postmortem samples of human PFC [221]. For example, *α*1 GABA_A_R subunit mRNA levels are very low perinatally and increase markedly until toddler ages, thereafter remaining consistently high through to adulthood [221]. In contrast, *α*2 subunit mRNA increased during the first postnatal months, decreasing subsequently until reaching mature levels at teenage years or young adulthood [221]. The mRNAs for *α*4 and *α*5 GABA_A_ subunits in human PFC showed a developmental pattern similar to that of *α*2 mRNA, whereas *α*3 subunit mRNA did not change significantly with age [221]. Postnatal expression of mRNA for *γ* and *β* GABA_A_R subunits similarly shows significant age-dependent changes, with *β*1 subunits showing a very early developmental decrease between neonate and infant ages, remaining constant thereafter, and *β*2 increasing somewhat later, between toddler and teenage years [222]. On the other hand, *γ*1 and *γ*3 subunit mRNA levels decrease with age during childhood and teenage years, whereas *γ*2 subunit mRNA levels decrease over the same period [222]. 

The developmental trajectories reviewed above suggest that similar processes underlie developmental changes in GABA_A_R-mediated synapses across various areas of human and rodent cortex, although further studies are necessary to properly compare developmental trajectories across species. A major difference between species is that the maturation of GABA-related markers in humans involves progressive change over one to two decades, whereas in rodents GABA synapse maturation appears to be complete within 3-4 postnatal weeks. Such difference suggests that the absolute time window during which activity and experience may influence GABA synapse development is markedly expanded in primate versus rodent brains. The prolonged period that may be necessary for the normal developmental tuning of the more complex circuitry of the primate cortex probably also prolongs the time window during which environmental factors can produce subtle developmental alterations that may contribute to the pathophysiology of schizophrenia.

## 6. Evidence Suggesting Alterations of GABA_**A**_R-Mediated Synaptic Transmission in the Cortex of Schizophrenia Patients

The hypothesis that a deficit in GABA_A_R-mediated transmission underlies cortical circuit dysfunction in schizophrenia is supported by convergent lines of evidence from post-mortem studies of the brain of subjects with schizophrenia [6]. Furthermore, such hypothesis is strengthened by the fact that one of the most reliably replicated findings in schizophrenia research is the decrease in GAD67 mRNA (for review, see [223]). Interestingly, equivalent measurements of GAD65 levels thus far failed to reveal alterations, suggesting that the role of GAD65 in GABA-mediated transmission maybe intact in schizophrenia. 

A remarkable recent study found that in schizophrenia several GABA-related transcripts, including those for GAD67, PV, GAT1, somatostatin, and the GABA_A_R subunits *α*1 and *δ*, show decreased levels in dorsolateral PFC as well as in the anterior cingulate, primary motor, and primary visual cortices [224]. Such conserved regional pattern of GABA alterations suggests that dysfunctional GABA neurotransmission contributes to multiple clinical features of schizophrenia including perceptual and motor deficits that could contribute to impaired cognitive function [11]. The disruption of PV expression across cortical areas confirms the previous findings and, moreover, suggests that alterations of PV-positive cells are central to the schizophrenia disease process, although the consequences of such decrease in PV are not completely understood. PV is a Ca^2+^ buffer that is present in nerve terminals of PV-positive neurons (Figure 1). Due to its slow kinetics of Ca^2+^ binding, PV is unable to bind intracellular Ca^2+^ before Ca^2+^ influx activates the Ca^2+^ sensor that triggers GABA release, because in PV-positive terminals Ca^2+^ influx is tightly coupled with GABA release [110, 225]. Interestingly, GABA release by single stimuli does not differ between PV-deficient and wild-type mice, but PV deficiency facilitates repetitive GABA release [226–228]. In synapses from PV-deficient mice, the amplitude of intracellular Ca^2+^ transients in nerve terminals is not affected, but their decay is slowed, indicating that PV normally accelerates such decay [227, 228]. Therefore, one possibility is that the decrease of PV in schizophrenia, instead of contributing to deficits, is a compensatory response to enhance GABA release in the face of decreased GABA synthesis. Alternatively, reduced PV levels may produce synaptic dysfunction via loss of some asynchronous release normally produced when Ca^2+^unbinds from PV, well after the presynaptic action potential ended [145]. A pathological loss of asynchronous GABA release by decreased PV levels, however, requires the existence of asynchronous release when PV is intact, a feature that is not observed in cortical PV-positive cells [104, 110], although it is found in PV-positive cerebellar interneurons [227]. 

Whereas schizophrenia may be associated with an increased density of *α2*-GABA_A_Rs at the AIS synapses from PVChCs [6], one study failed to detect significant changes in total tissue levels of *α*2-GABA_A_R mRNA [221]. One possibility is that changes in *α*2-GABA_A_Rs are synapse- or layer-specific and perhaps found exclusively at AIS synapses in superficial cortical layers, as initially reported [229]. Consistent with this interpretation, laminar analysis of mRNA levels for GABA_A_R subunits in the cortex of subjects with schizophrenia revealed significantly increased levels of mRNA for *α*2 GABA_A_R subunits exclusively in layer 2 [230]. Moreover, the same study revealed lower levels of *α*1 GABA_A_R subunit mRNA in layers 3 and 4 [230], which is consistent with a decrease in total tissue levels of *α*1 GABA_A_R subunit mRNA observed using quantitative PCR [224]. Given that *α*1 subunit-containing GABA_A_Rs constitute about 60% of the total GABA_A_Rs in adult brain [231], it is possible that *α*1 subunit mRNA is significantly more abundant than that for *α*2 subunits, thus increasing the chance of detecting changes of total tissue *α*1 mRNA levels in schizophrenia. Importantly, a decrease in *α*1 subunits in schizophrenia is consistent with weaker synaptic transmission from PVBCs, since *α*1 subunit-containing GABA_A_Rs are predominant at synapses from mature PVBCs [33, 41, 101, 102]. 

In addition to GABA_A_R levels, the strength of the postsynaptic response to GABA depends on the driving force for the GABA_A_R current which is determined by its reversal potential *E*_GABA_A__. As *E*_GABA_A__ depends on chloride extrusion by KCC2 and chloride uptake by NKCC1 [43], a recent study examined mRNA expression for both chloride transporters in the cortex of subjects with schizophrenia [232]. Interestingly, KCC2 and NKCC1 transcript levels were not altered in subjects with schizophrenia; however, transcripts for two kinases (OXSR1 and WNK3) that strongly regulate KCC2 and NKCC1 activity in opposite directions, are overexpressed in schizophrenia [232]. If increased levels of OXSR1 and WNK3 mRNA actually represent increased kinase activity, then the chloride gradient across the postsynaptic membrane may be decreased, resulting in an *E*_GABA_A__ significantly more depolarized than normal [232]. Since normally *E*_GABA_A__ varies with cell type and subcellular compartment, understanding the consequences of alterations in chloride transport requires a detailed quantitative analysis of protein localization and activity, a challenging task in this case, given that postmortem interval effects alter the integrity of some of these proteins [232]. 

Direct demonstration that GABA-mediated synaptic inhibition is decreased in the cortex of subjects with schizophrenia is challenging. Interestingly, magnetic resonance spectroscopy (MRS) was recently applied to noninvasively measured GABA concentration in human neocortex and determined whether a decrease of GABA is observed in schizophrenia. MRS does not distinguish extracellular GABA from transmitter stored in particular cellular compartments or cell types and also lacks adequate temporal resolution but nevertheless reveals activity-dependent changes in GABA levels. For example, acute psychological stress which elevates subjective anxiety produces a short-term decrease in GABA concentration in human dorsolateral PFC that can be detected with MRS [233]. Combining MRS and EEG in the same subjects, the relations between brain GABA content and oscillatory neural activity in schizophrenia may be explored. Interestingly, in normal human subjects, GABA concentration measured in visual cortex with MRS was positively correlated with the strength of gamma oscillations induced by visual stimulation [234]. Moreover, interindividual variation in GABA concentration determined by MRS in visual cortex was correlated with variability of performance in a visual stimulus orientation discrimination task that induces gamma oscillations [235]. 

Measurements of tissue GABA concentration with MRS may help in clarifying the relations between GAD67 levels, gamma oscillations, and cognitive performance in schizophrenia. One such study did not detect differences in GABA concentration in the anterior cingulate cortex of schizophrenia versus control subjects, whereas GABA concentration was apparently decreased by antipsychotic medications [236]. Another MRS study reported reduced GABA concentration in basal ganglia but not frontal cortex of schizophrenia patients [237]. In patients with relatively low antipsychotic exposure, MRS revealed a significant reduction of GABA concentration in visual cortex that did not covary with medication dosage but was correlated with behavioral abnormalities in a visual surround-suppression task thought to depend on GABA-mediated inhibition [238]. Moreover, a longitudinal study of early-stage first-episode schizophrenia patients showed that 6 months of treatment with atypical antipsychotics did not change GABA concentrations measured with MRS in frontal and parietal lobe cortices nor in basal ganglia [239]. In contrast, MRS revealed elevated GABA concentration in anterior cingulate and parietal cortex of subjects with chronically treated schizophrenia compared to control subjects [240]. The MRS findings suggesting that antipsychotics may change brain GABA concentration highlight the importance of addressing the effects of confounding factors such as medications [241]. Significantly, both postmortem studies in humans and experimental studies in animals have failed to show an effect of antipsychotic medications on GAD67 mRNA levels [224, 242, 243]. The data from MRS studies therefore underscore the importance of combining neurochemical, electrophysiological, and behavioral assessment, given the large interindividual variability in bulk GABA concentration, gamma activity levels, and behavioral performance. Instead of or in addition to medication effects, the large variability in cortical GABA content measured with MRS in human cortex may be explained by the effects of genetic variants in the *GAD1* gene that may differentially confer risk of schizophrenia [244].

## 7. Conclusions

The findings reviewed here suggest that alterations of GABA transmission produce cognitive deficits in schizophrenia by altering the circuit mechanisms of gamma oscillations. These observations suggest a molecular and cellular basis for the development of new therapeutic interventions [245]. Importantly, the proposal that GABA alterations are linked to altered gamma oscillations and cognition is supported, at least in part, by animal model studies showing that producing a functional loss of GABA-mediated inhibition diminishes gamma oscillations [246] and impairs cognitive function [247, 248]. Whereas work in animal models is essential, the difficulty of capturing in animals the complexity of behavioral alterations in a uniquely human disorder may explain the relative lack of success in developing drugs to treat schizophrenia compared with other disease areas [4, 5, 245]. Interpretation of studies in human subjects based on comparisons between healthy controls and patients is complicated as well, given that schizophrenia versus control differences may actually reflect pathogenesis but also could represent compensatory changes or effects of confounding factors [4, 241]. For example, the effects of producing a transient deficit in GABA_A_R-mediated signaling were tested recently in human subjects [249] using iomazenil, a compound that binds to the benzodiazepine site of GABA_A_Rs and negatively modulates the effects of GABA (i.e., an inverse agonist). Consistent with dysfunctional GABA_A_R signaling in schizophrenia, iomazenil produced perceptual deficits and psychotic symptoms in schizophrenia patients at doses that did not affect healthy control subjects [249]. However, the schizophrenia patients in such study chronically received antipsychotics and anxiolytics, raising the question of whether an interaction between acute iomazenil and chronic medications influenced such findings. 

Preliminary tests of the idea that enhancing GABA_A_R signaling improves behavioral and electrophysiological measures in subjects with schizophrenia were conducted in two recent studies evaluating the effects of MK-0777, an *α*2/*α*3 GABA_A_R-preferring positive allosteric modulator [47]. In one study, randomized administration of MK-0777 or placebo in double-blind fashion improved performance of schizophrenia patients in a cognitive control task, simultaneously increasing gamma oscillation power in frontal cortex [250]. In contrast, the second study did not find significant effects of MK-0777 compared with placebo in the performance of patients in a battery of tests designed to assess cognitive function in schizophrenia [251]. The inconsistent beneficial effects of MK-0777 administration might be explained by the fact that MK-0777 is a partial agonist at *α*2/*α*3 subunit-containing GABA_A_Rs with only about 10–20% potency compared to a full agonist [48]. Thus, one possibility is that more potent *α*2/*α*3 benzodiazepine site agonists need to be employed. Such drugs should also be more selective because, compared with placebo, MK-0777 had a tendency to produce sedation and somnolence [250, 251], which could mask improvements in cognitive performance. Importantly, the *α*2/*α*3 GABA_A_R modulator MK-0777 was selected based on the compelling rationale that inputs from PVChCs onto the AIS show important alterations in schizophrenia [83] and that, depending on cortical layer, such inputs involve *α*2-GABA_A_Rs or *α*3-GABA_A_Rs [101, 201, 252]. However, whether or not PVChCs play a role in production of gamma band synchrony remains unclear [82], and so it is possible that PVChC alterations in schizophrenia produce cognitive deficits unrelated to dysfunctional gamma band synchrony. Therefore, further information from both basic and clinical research studies is necessary to further assess the effectiveness of *α*2/*α*3 GABA_A_R modulators for treatment of gamma synchrony-related cognitive deficits in schizophrenia. Indeed, basic research studies continue to provide insight into the role of specific subtypes of GABA neurons on inhibition-mediated cortical network oscillations [127], and molecular pharmacology studies are identifying novel compounds acting at different sites within the GABA_A_R receptor complex [44, 47, 253]. 

Importantly, a potential role of GABA-mediated neural synchrony in cortical circuits is to enable spike-timing-dependent plasticity, indirectly modifying the strength and stability of excitatory synaptic connections [254]. Whether spike-timing-dependent plasticity at glutamate synapses is impaired in schizophrenia is not yet clear [12], but it is possible that the decrease of dendritic spine density in pyramidal neurons in schizophrenia is due to glutamate synapse loss produced by altered plasticity mechanisms [241]. If neural synchrony-dependent glutamate synaptic plasticity is dysfunctional in schizophrenia, then cognitive enhancement behavioral therapies that involve learning paradigms may help in preventing or reversing the consequences of altered circuitry on the induction of synaptic plasticity. Interestingly, cognitive enhancement behavioral therapy was recently shown to improve cognition and prevent gray matter loss in schizophrenia [255]. Potentially, combining cognitive therapies with pharmacological treatment that boosts otherwise weakened neural synchrony may constitute an effective treatment intervention in schizophrenia, as for other psychiatric disorders [256].
